# Society for the Study of Comparative Psycology, of McGill University, Montreal

**Published:** 1892-02

**Authors:** 


PROCEEDINGS OF THE SOCIETY FOR THE STUDY OF COMPARATIVE PSYCHOLOGY, IN CONNECTION WITH THE FACULTY OF COMPARATIVE MEDICINE AND VETERINARY SCIENCE OF MCGILL UNIVERSITY, MONTREAL, FOR 1890–91.

Some ten meetings were held, at most of which, in addition to routine and special business, papers were read.

At the first meeting, Oct, 8, 1890, officers were elected and new members proposed. At the second, on Oct. 16, Prof. Girdwood delivered the opening address, which has appeared in full in the *Montreal Gazette*. At a subsequent meeting. on Oct. 29, papers were read which went to show that the simple explanations usually given of the acts of the lower animals were inadequate. A large number of the members present took part in a most interesting discussion which followed. It was shown that various animals, especially dogs and horses, possessed wonderful powers of memory. They could return home by ways they had never traveled in so short a time as to make the whole performance almost beyond our conception So much so was this the case that Darwin had suggested that some of the lower animals were possessed of a special faculty not shared by man. One member believed that a great deal of the behaviour of animals under such circumstances was due to a "sense of uncongeniality" in the surroundings, which led them to move, till they met the conditions that were favorable.

The President, Prof. Wesley Mills, thought this suggestion a very good one, but he believed that there were landmarks and various recognizable appearances and phenomena which animals do (and which man might, if he would look more carefully) note and use, as, indeed, men living in a condition of primitive civilization, analogous to the ways of the lower animals, do employ.

Prof. Penhallow reported, through the President, an ingenious use by a horse of a mechanical means to accomplish an end.

A member gave a thrilling account of his escape from death and that of a companion, during a blizzard in the Northwest, owing, apparently, to the intelligence of the horses they drove, which they were obliged finally to abandon to their own guidance.

Another member stated that he had driven a pony thirty-five miles along a road with forty-two turns, and when returning at night it was so dark that he had to leave the horse to himself to pick the way, which he did successfully, though he had never been over this road before.

At the last meeting a paper was read on the psychic nature of the horse. It was a clear, neat and convincing discussion of the subject, and showed, among other things, that a horse of a certain temperament may be made to act as if vicious by one who does not use him well Such behavior on the part of the animal was in reality a protest against the cruelty of the attendant.

The President related an instance which had happened a few days before with the dogs from his own kennel. A fine old pointer had detected the presence of a grouse by scent and pointed it, backed by a young Irish setter ; but another setter coming up and not catching the scent, etc., had driven away the bird, whereupon the old pointer set upon it and chastised it for its folly.

The second paper of the evening was on the dog. The conclusions were well supported by facts observed by the writer of the paper. He mentioned the case of a spaniel that killed sheep, and, before coming home, would wash in a pond and return to his master's by a devious course. The writer also made reference to the case of a dog that was very ferocious towards him till he had assisted in relieving the dog after an accident, when the animal manifested gratitude and never forgot the kindness.

At a meeting of the Association on Nov. 26, the President, Dr. Wesley Mills, in the chair, a paper was read by Mr. McCaulay on "Instinct and Reasoning," and another by Mr. T. B. McDonald on the "Powers of Discrimination in the Dog and Horse," a third followed by Mr. D. M. McDonald on the psychic nature of the cow and the dog.

The first paper was a discussion of the limits of instinct and reason in animals. The other two papers considered the light in which certain striking facts observed by themselves were to be viewed. The President pointed out that after all in this as in other sciences, terms but inadequately represent the facts. It was impossible to analyse cases so perfectly that we could say that this or that act was due wholly to either instinct or reason, at least in a multitude of cases. Animals, like men, acted under a variety of impulses and motives; and in not a few instances their instincts were as much modified as our own. This remark was especially applicable to our domestic animals. In the case of Mr. McDonald's dog that so sympathetically watched the packing of his owner's trunk we had a close resemblance to what might be observed in a rather young child. The power to appreciate articulate language made the great difference. It was, on the whole, wise to recognize the great resemblance of their psychic processes in our domestic animals to our own as well as the differences. Formerly too much stress was laid on the latter.

The Executive reported that 400 copies of Dr. Girdwood's address had been printed and would be distributed to those having a special interest in such subjects.

The next meeting was held in the usual place on Dec. 10, the President in the chair.

Mr. Miller read a paper on "The Extent to which Animals can Reason." He illustrated his position by the behavior of a single pair of hounds that he frequently saw hunting foxes. They evidently acted in concert to cut off ground and save their energies alternately, so that one hound was always in full chase. He mentioned the case of a mule that had been in a loose box in a stable for an injury to its leg. After the lapse of months it again was injured by a nail passing into the foot. When turned into the stable, instead of going to its usual place, it went once more to the loose box it had occupied when a patient before. Was this not reasoning? The writer and others referred to the intelligence shown by animals in the time and method they adopt in breaking into fields. The latter topic gave rise to considerable discussion, and was pretty well threshed out.

Mr. McCrank read a paper on "The Psychic Status of the Dog." The writer made many comparisons between man and the dog, giving his facts and reasons, from which it appeared that the dog was not always and in all respects the lower animal. He had made a special study of a dog of his own, a cross bred Skye terrier, that showed not only great aptitude in learning, but much individual resource in meeting various exigencies. This dog seemed to recognize clearly the time of day in the way he attended to certain duties assigned to him, such as bringing home the cattle, etc. In one instance he had been defended by an old sow when being worried by another dog. He ever afterwards exhibited gratitude towards this animal. The writer clearly proved that his dog could learn the use of words, and analyzed the circumstances, showing that this dog, at all events, compared favorably in this respect with the average child, and remembered his lessons even better. He showed that he could distinguish between the appellation of his brother "Pat"

and his father "Pa;" for while "Pat" teased him "Pa" was kind and the dog acted differently when these words were mentioned.

The President was of the opinion that the writer's demonstration of his thesis, especially as regards the capacity of the dog to understand language, was the most conclusive that had as yet been brought before the association; and while the paper was somewhat extreme, it was conceived in a good spirit and written in a scientific manner that made it the most valuable he had heard.

A meeting of the Society was held January 19th, the President in the chair. Mr. Simpson was elected Secretary–Treasurer instead of Mr. Sturrock, who was obliged to resign, owing to illness in his family.

The first paper of the evening was read by Mr St. Louis, and had special reference to canine psychology. He owned a collie dog that had been punished for barking when his master came home at night. He soon learned to distinguish the sound of his owner's wagon, and even of his horse's hoofs, for he never after barked when he arrived, whether riding or driving. This indicated both general intelligence and a nice power of discrimination by ear. This dog would also bring any one of the cows belonging to the farm when sent for it by name. The writer of the paper explained how the names of the cows had been taught the dog during the winter season when they were kept in the farmyard.

Mr. St. Louis also gave an account of a dog bred from a collie and a Newfoundland, that would carry his master's dinner, including the tea to him, from home. On one occasion he spilled the tea on the way, and of his own accord came back and held up the empty can. On being given more he made extra speed, so that he arrived only ten minutes later than his usual time. The same dog would fill the wood-box at night with either hard or soft wood, as told.

This paper gave rise to some discussion, the President pointing out that whether dogs and other of our domestic animals had a more acute sense of hearing than man or not, they certainly were quicker to perceive the vibrations in solid bodies, as evidenced, among other things, by their anxiety and alarm prior to earthquakes, owing, no doubt, to their perception of the slight oscillations of the earth unnoticed by man, but which delicate apparatus had shown existed.

Mr. Twombly read a clear and thoughtful paper on the general principles that underlie comparative psychology, showing that, naturally, man's psychic nature was the standard by which he was obliged to judge all other minds. The different degrees of development of the nervous system, and especially of the brain, was a good guide as to relative intelligence. The paper does not well admit of condensation.

At a meeting held on February 4th, 1891, a paper was read by Mr. Townsend in which an attempt was made to draw the line between instinct and reason, the history of a collie dog being given, whose conduct could not be explained either by instinct or by reason alone, and it seemed a clear case to illustrate both. He also gave an account of concerted action between a bull terrier and a collie dog. On one occasion he had attempted to remove some hens that were accustomed to seek shelter under an old building where a hog habitually made its bed. The pig manifested anger and approached him with open mouth It seemed in this case as if the hog had taken the hens under its protection, and this corresponded with an instance that another member had brought to their notice at a recent meeting.

The President said that he considered that the good qualities and intelligence of the pig tribe were underrated.

The next paper was by Mr. Watson, on "Sheep-worrying by Dogs." This was a valuable paper, and all the more so, as it was based entirely on his own observations. He explained how a pure bred collie bitch of his own and one of her puppies had taken to chasing sheep, only after hares which they were accustomed to hunt of their own accord, became scarce. Both these animals were very intelligent and readily taught. Mr. Watson felt satisfied that the dogs at first chased sheep simply for amusement. After they had once formed the habit it seemed impossible to cure them of it, as, however long they were confined, when let loose they would at once seek for some animal to chase, it might be horses, hares or sheep.

The subject was well discussed. One gentleman gave an account of dogs learning to worry sheep when the former were neglected. Several members believed that sheep were often killed more by fright than the lacerations or other injuries they received. It seemed likely that in some instances the blood was sucked from the veins of the neck. All dogs that worried sheep seemed to be, almost without exception, unusually cunning in evading detection. The President thought the remedy for sheep-worrying was its prevention by municipalities requiring all dogs to be confined during the night. A dog was useful as a watch, not by his bite, but by his bark, and it was not necessary to allow him at large ; indeed, that was a nuisance.

At a final meeting an excellent paper was read by Mr. Simpson on "The Feelings of Animals," and one by Mr. McNaughton on "The Intelligence of the Pig."

At a special meeting held on December 30th, the diploma of honorary fellowship of the Society was conferred on those that had fully complied with all the conditions. The Society then adjourned to meet in October.

